# Platelet and Leukocyte Concentration in Equine Autologous Conditioned Plasma Are Inversely Distributed by Layer and Are Not Affected by Centrifugation Rate

**DOI:** 10.3389/fvets.2020.00173

**Published:** 2020-05-12

**Authors:** Alexandra V. Radtke, Margaret B. Goodale, Lisa A. Fortier

**Affiliations:** Department of Clinical Sciences, College of Veterinary Medicine, Cornell University, Ithaca, NY, United States

**Keywords:** platelet rich plasma, PRP, equine, ACP, leukocyte reduced, P-PRP

## Abstract

**Background:** Platelet rich plasma (PRP) is used extensively in equine regenerative medicine. Differences in preparation protocols give rise to significant variability in the cellular composition of PRP making it very difficult to establish a standard of care in the field. This study aimed to optimize the preparation protocol for leukocyte-reduced PRP (P-PRP).

**Methods:** Blood (100 mL) was collected from horses (*n* = 5) and divided into 2 purple top EDTA tubes and 6 (15 mL) double syringes^a^ with a final concentration of 10% acid citrate dextrose anticoagulant. Six double syringes^a^ were collected from each horse; PRP samples were prepared in duplicate and centrifuged at 1,100 rpm (188 × g), 1,300 rpm (263 × g), or 1,500 rpm (350 × g). Duplicates were subjected to +/– braking at the end of centrifugation. The total volume of PRP generated was measured and divided into thirds. Each third (top, middle, and bottom) were drawn off separately using the inner (6 mL syringe) and placed in purple top EDTA tubes. Automated complete blood counts were performed on all peripheral whole blood and PRP samples.

**Results:** The concentration of leukocytes was higher in the bottom layer of PRP compared to the top and middle layers (*p* < 0.0001). The concentration of platelets was slightly lower in the bottom layer of PRP than the middle layer (*p* = 0.02). Centrifugation braking increased the leukocyte concentration in the top (*p* = 0.03) and middle layers of PRP (*p* = 0.001). Centrifugation rate had no effect on the cellular composition of PRP (*p* = 0.1–0.6).

**Conclusions:** Because layer of plasma affected both platelet and leukocyte concentrations in PRP, the most important modification for the current single spin, double syringe, plasma based PRP preparation protocols is to exclude the bottom 1/3 layer of PRP.

## Introduction

Musculoskeletal injuries are common in equine athletes and, due to the poor intrinsic healing capabilities of cartilage and tendons, present a significant clinical challenge (1, 2). PRP has been used extensively by equine practitioners and specialists to promote healing by delivering anabolic growth factors to damaged tissue (3–6). In human medicine, there is considerably more robust evidence for the application of PRP in musculoskeletal injury with several level one studies supporting the use of PRP for tendinopathies and osteoarthritis (7–16). There is however, remaining debate regarding the optimal PRP preparation. Specifically, controversy exists regarding the effects of leukocytes within PRP. Some studies suggest a beneficial effect including increased growth factor and cytokine release as well as increased antibacterial and immunologic resistance (17). Other studies however suggest a negative effect including increased inflammatory cytokines and catabolic mediators release leading to the degradation of tendon, ligament, muscle, and cartilage (18, 19). Recent evidence suggests that minimizing leukocyte concentration in PRP is more important than maximizing platelet concentration for decreasing inflammation and enhancing matrix gene synthesis (18–23). Broadly, plasma-based centrifugation methods produce leukocyte-reduced PRP (P-PRP) in contrast to buffy coat based methods which concentrate both platelets and leukocytes (L-PRP) (24, 25).

Many techniques exist for the preparation of P-PRP in either a liquid or solid fibrin form (18, 26–29). For veterinary applications, liquid injectable preparations of PRP are most commonly used. Preparation protocols for P-PRP differ by centrifugation rate (188–350 × g), centrifugation time (4–6 min), and braking at the end of centrifugation (26). Each of these factors can affect the platelet and leukocyte composition of PRP and therefore the clinical application of PRP for therapeutic application.

Most commercially available PRP systems were designed for use with human blood, posing a challenge for equine practitioners due to the lower average hematocrit in horses (33%) compared to humans (45%) (30, 31). Using the double syringe system^a^ as an example, the lower equine hematocrit leads to generation of an excessive volume of PRP (~9 mL of PRP), which is greater than the maximum 6 mLs the double syringe system^a^ is designed to produce. When the top 6 mLs of PRP are withdrawn, the 3 mLs of PRP closest to the packed red blood cell layer remain. Conventional wisdom would suggest that these remaining 3 mLs of PRP would contain more platelets than the upper 6 mLs simply due to platelet sedimentation during centrifugation, but the platelet and leukocyte concentrations have not been measured in the various levels of ACP. The objective of this study was to assess how centrifugation factors including; centrifugation rate, centrifugation braking, and layer of plasma affected the distribution of platelet and leukocyte concentration in PRP. This information will help establish a more standardized protocol for single-spin P-PRP methods.

## Materials and Methods

### Study Overview

**Figure 1:**
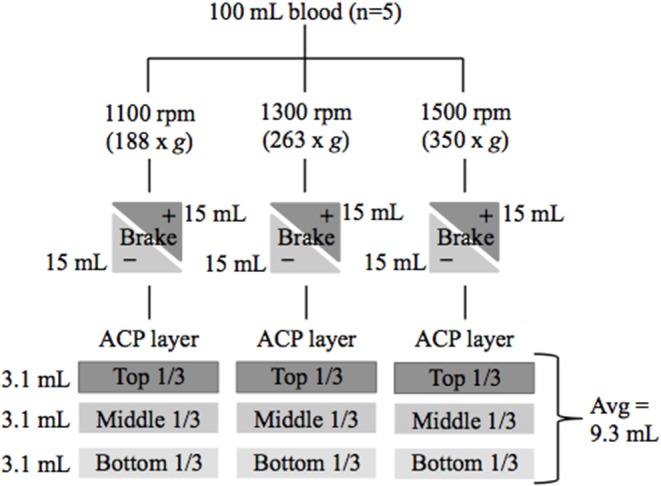
Flow chart of study design. Blood was collected from 5 healthy horses. Platelet rich plasma (PRP) was generated using 6 centrifugation techniques with variations in centrifugation rate and braking. PRP was divided into three layers and CBCs were performed to determine cellular composition. rpm, revolutions per minute; g, g force; (+) brake was applied or (–) brake was not applied at end of centrifugation.

Platelet rich plasma (PRP) was generated from 5 healthy mature horses (3 geldings and 2 mares) of various breeds (2 Thoroughbreds, 2 Warmbloods, and 1 Quarter Horse) with a mean age of 8 years (range: 4–21 years). Three different centrifugation rates, each with and without a brake were used to generate PRP. The top, middle, and lowest 1/3 of each PRP preparation were analyzed for platelet and leukocyte concentration (Figure 1). This study was approved by the Institutional Animal Care and Use Committee of Cornell University.

### Platelet Rich Plasma

Blood (100 mL) was collected and divided into 2 purple top EDTA tubes and 6 (15 mL) double syringes^a^ containing acid citrate dextrose (ACD) anticoagulant to a final concentration of 10%. To test the effect of centrifugation rate on the distribution of leukocytes and platelets throughout the plasma, PRP was prepared in duplicate with centrifugation at 1,100 (188 × g), 1,300 (263 × g), or 1,500 rpm (350 × g), each for 5 min using a Hettich Rotofix 32 centrifuge. One of the duplicate syringes at each centrifugation rate was subjected to braking (25 s deceleration using the rotor brake) at the end of centrifugation and the other was not (9 min deceleration using gravity/friction).

After centrifugation, the total volume of PRP generated was measured and recorded. For each double syringe^a^, the total PRP volume was divided into thirds (ex. total volume 9 = 3 mL per third). The PRP was extracted and placed into purple top tubes one layer at a time (ex. 3 mL) from the 15 mL double syringe^a^ using the inner (6 mL) syringe per manufacturer's instructions. These samples were labeled as top layer (farthest from RBCs), middle layer, and bottom layer (closest to the RBCs). Complete blood counts (CBCs) were performed on both the peripheral whole blood and PRP samples by Cornell University Clinical Pathology using a Bayer ADVIA 2120 automated analyzer.

### Statistical Methods

For measures of PRP composition (platelets, total leukocytes, segmented neutrophils, lymphocytes, and monocytes), a mixed-effect model was fitted to the data. Horse was treated as a random effect and centrifugation rate (188, 263, or 350 × g), brake (+/–), and layer (top, middle, or bottom 1/3) were treated as fixed effects, with interaction terms for centrifugation rate^*^brake, brake^*^layer, and layer^*^centrifugation rate. Platelet and leukocyte fold change was calculated by dividing the concentration of cells in PRP by the concentration of cells in peripheral whole blood. PRP layer was evaluated by comparing the top, middle and bottom layers (~3 mL aliquots). Centrifugation rate and braking were evaluated by comparing the average [(top + middle + bottom layers)/3] of each sample vs. samples of varying centrifugation rates and/or braking status. Statistical analysis was performed using a statistical software program with *p* < 0.05 considered significant. Effect size was calculated for significant findings.

## Results

A total of 95 CBCs were performed on 30 PRP and 5 peripheral whole blood samples. The average amount of PRP produced was 9.25 mL (range 7.0–10.0 mL) per double syringe^a^. Data from one horse was excluded from the statistical analysis of platelet fold change due to platelet clumping in the whole blood sample (flagged in automated CBC report and confirmed on blood smear) making a fold change calculation inaccurate. However, for leukocyte concentration, leukocyte fold change, and platelet concentration data from all 5 horses were used.

### Platelet Concentration

Platelet concentration was affected by the layer of PRP, but not by centrifugation rate or braking. Platelet concentration varied between the 3 layers of PRP (Table 1), with the bottom layer of PRP having a slightly lower platelet concentration (*p* = 0.02) and lower platelet fold change (*p* = 0.04) than the middle layer. There was no difference in platelet concentration between the top and bottom layers of PRP.

**Table 1:** Data table representing whole blood and PRP samples.

			**Platelet concentration** **(thou/μL)**	**Platelet fold change**	**Leukocyte concentration** **(thou/μL)**	**Leukocyte** **fold change**	**Monocyte concentration** **(thou/μL)**	**Lymphocyte concentration** **(thou/μL)**	**Neutrophil concentration** **(thou/μL)**
Peripheral blood			120		5.4		0.2	1.8	3.2
PRP	Centrifugation factor							
	PRP layer	Top	185	1.6	1.0	0.2	0.03	0.6	0.3
		Middle	200	1.8	1.7	0.3	0.07	1.0	0.6
		Bottom	174^*^	1.5^*^	6.9^*^^†^	1.2^*^^†^	0.3^*^^†^	2.8^*^^†^	3.7^*^^†^
	Centrifugation Rate	1,100 rpm (188 × g)	186	1.6	3.9	0.7	0.2	1.8	1.8
		1300 rpm (263 × g)	191	1.7	2.4	0.4	0.1	1.1	1.1
		1,500 rpm (350 × g)	181	1.6	3.4	0.6	0.2	1.4	1.7
	Centrifugation braking	(+) Brake	189	1.7	3.9^‡^	0.7^‡^	0.2	1.7	1.8
		(–) Brake	183	1.6	2.5^‡^	0.5^‡^	0.1	1.2	1.2

Concentrations represent the mean average of all samples (N = 4 horses for platelet concentration and fold change; N = 5 horses for Leukocyte, Monocyte, Lymphocyte, and Neutrophil concentration and Leukocyte fold change). Asterisk (^*^) represents a p < 0.05 between the bottom and middle layers of PRP; the dagger (^†^) represents a p < 0.05 between the top and bottom layers of PRP; and the double dagger (^‡^) represents a p < 0.05 between (+) and (–) centrifugation brake.

Variations in centrifugation rate and braking at the end of centrifugation did not affect platelet concentration in PRP. There was little variation in platelet concentration (*p* = 0.60) or platelet fold change (*p* = 0.40) between PRP generated at 188, 263, 350 × g. The greatest difference in platelet concentration was between the 263 and 350 × g groups with the 350 × g group containing an average of 2% more platelets than the 263 × g group. Centrifugation braking had no effect on platelet concentration (*p* = 0.40) or platelet fold change (*p* = 0.40). Platelet concentration was increased by <2% when braking was used at the end of centrifugation.

### Leukocyte Concentration

In direct contrast to the increased platelet concentration in the middle layer of PRP, leukocyte concentration was increased in the bottom layer. Both the total leukocyte concentration (*p* < 0.0001) and leukocyte fold change (*p* < 0.0001) were increased in the bottom layer of PRP compared to the middle and top layers (Table 1). The majority (72%) of leukocytes measured in the PRP were in the bottom layer. The bottom layer of PRP was leukocyte concentrated with a leukocyte fold change of 1.2, whereas the top and middle thirds were leukocyte reduced with a 0.2- and 0.3-fold change, respectively. All three leukocyte subtypes; monocytes (*p* < 0.0001), lymphocytes (*p* < 0.0001), and neutrophils (*p* < 0.0001), were increased in the bottom layer of PRP (Table 1). Monocyte concentration was 5.1 and 12.7 times higher in the bottom layer of PRP than in the middle and top layers, respectively. Lymphocyte concentration was 2.7 and 4.5 times higher in the bottom layer of PRP than in the middle and top layers, respectively, and neutrophil concentration was 6.6 and 10.7 times higher in the bottom layer of PRP than in the middle and top layers, respectively.

Variations in centrifugation rate did not affect leukocyte concentration (*p* = 0.10) or leukocyte fold change (*p* = 0.09) in ACP (Table 1). Leukocyte concentration was 1.4 and 1.6 times higher in PRP generated at 350 and 188 × g than at 263 × g, but this difference was not significant (*p* = 0.10).

Braking at the end of centrifugation increased leukocyte concentration (*p* = 0.04) and leukocyte fold change (*p* = 0.04) in PRP (Table 1). Braking increased the leukocyte concentration by ~1,500 (leukocytes/μL) in each layer of PRP leading to a 3.9, 4.6, and 1.1 times increase in leukocyte concentration in the top, middle, and bottom layers of PRP, respectively. Leukocyte concentration increased significantly in the top (*p* = 0.03) and middle (*p* = 0.001) layers of PRP when the brake was applied, however the increase was not significant in the bottom layer (*p* = 0.8). It is important to note that although braking increased leukocyte concentration in PRP, the top and middle layers remained leuko-reduced (lower concentration compared to starting blood sample) regardless of braking.

## Discussion

The data from this study suggests that the top 2/3 of PRP produced using the double syringe, single-spin plasma-based method have the optimal platelet: leukocyte ratio and platelet dose for treatment of tendonitis and osteoarthritis. A centrifugation rate between 188 and 350 × g can be used to consistently produce P-PRP using the single spin plasma-based method. Centrifugation braking will increase leukocyte concentration throughout the PRP layer, however the top 2/3 will remain leuko-reduced.

Despite the fact that plasma-based systems concentrate platelets in the plasma portion of the sample, many practitioners believe that the layer closest to the buffy coat contains the highest concentration of platelets and many try to include this layer or even a small portion of the buffy coat into their sample to maximize platelet retrieval. The finding from this study contradict this line of thinking. Hematologic evaluation revealed significant variations in the cellular composition of PRP when divided into three layers. Platelets in the top and middle layers of PRP were increased 1.6 and 1.8-fold over whole blood whereas platelets in the bottom layer were only increased by 1.4-fold. This represented a significant difference in platelet fold change suggesting that the majority of concentrated platelets were in the top 2/3 of PRP (away from the buffy coat).

In regenerative medicine, the “more is better” concept has dominated when considering total platelet dose. Recent investigations have shown that improvement in tissue healing is achieved with low to moderate platelet doses and the amount of improvement in tissue healing plateaus with increasing platelet doses. Multiple level one studies have reported successful outcomes using 0.9–9.0 × 10^9^ platelets/dose (7, 8, 12). In the present study, an average platelet dose of 1.4 × 10^9^ was produced in just the top 2/3 of PRP. This dose is well within the range necessary to improve tissue healing; therefore, it would be acceptable to exclude the bottom 1/3 layer of PRP.

Centrifugation is a key factor in PRP preparation as it separates blood components based on their density gradients. Platelets are the lightest, followed by leukocytes, and then RBCs, which are heaviest (32). Therefore, relative centrifugal force (RCF) must be appropriate to obtain good separation of platelets from other blood cells. In this study, a range of low centrifugation rates and a short duration of centrifugation time resulted in the reliable and consistent production of P-PRP. We compared a range of slow centrifugation rates, 188, 263, and 350 × g, which are standard for the single spin plasma-based method protocol. Our results showed that there was no difference in the cellular composition of PRP between these groups. Previous studies reported a 1.2–2.5-fold increase in platelet concentration and a 0.06–0.2-fold decrease in leukocyte concentration using the standard (350 × g, 5 min) ACP protocol (33–35). PRP generated in our study had an average of 1.5-fold increase in platelet concentration (with no significant difference between centrifugation rate groups). This is in line with previously reported values. The PRP produced in our study also had an average of 0.6-fold decrease in leukocyte concentration. This does not correlate with the results of previous studies, however our study included PRP from the bottom 1/3 layer of plasma (closest to the buffy coat) which had a 1.2-fold increase in leukocyte concentration vs. the middle and top layers which had a 0.2 and 0.3 fold decrease in leukocyte concentration, respectively. Therefore, the composition of PRP generated during our study is in line with previously reported values when the bottom 1/3 layer is excluded. We concluded that a slow centrifugation rate, between 188 and 350 × g, can be used to consistently produce P-PRP using the single spin plasma-based method.

Braking at the end of centrifugation was also evaluated in this study. We found that the use of a rotor brake (which reduces centrifugation time by about 9 min) had no impact on platelet concentration in PRP but caused a slight increase in leukocyte concentration in the top and middle layers of PRP. This is consistent with the results of previous studies which showed that rapid deceleration induced turbulence and mixing of WBCs into the PRP (32). However, the amount of leukocyte contamination in the top and middle layers of PRP after braking was minimal—with the end product continuing to be significantly leuko-reduced at 0.2- and 0.3-fold decrease in leukocyte concentration in the top and middle layers, respectively. Therefore, the use of a rotor brake at the end of centrifugation does not significantly alter the cellular composition of P-PRP.

The results of this study suggest that differences in the cellular composition of PRP due to various centrifugation and preparation factors should be considered when determining the optimal P-PRP protocol. Layer represented the most significant factor influencing the composition of PRP. When seeking to produce P-PRP, the top and middle layers have the best cellular composition with a platelet concentration in the range suggested for improved tissue healing and an 80% reduction in leukocyte concentration.

## Data Availability Statement

The raw data supporting the conclusions of this article will be made available by the authors, without undue reservation, to any qualified researcher.

## Ethics Statement

This study was approved by the Institutional Animal Care and Use Committee of Cornell University.

## Author Contributions

AR and MG executed the study. All authors contributed equally to study design, data review, and manuscript preparation.

## Conflict of Interest

LF is a consultant to Arthrex, Inc. whom donated the ACP syringes for the study. The remaining authors declare that the research was conducted in the absence of any commercial or financial relationships that could be construed as a potential conflict of interest.
